# The monomeric form of *Neisseria* DNA mimic protein DMP19 prevents DNA from binding to the histone-like HU protein

**DOI:** 10.1371/journal.pone.0189461

**Published:** 2017-12-08

**Authors:** Ming-Fen Huang, Shin-Jen Lin, Tzu-Ping Ko, Yi-Ting Liao, Kai-Cheng Hsu, Hao-Ching Wang

**Affiliations:** 1 Graduate Institute of Translational Medicine, College of Medical Science and Technology, Taipei Medical University, Taipei, Taiwan; 2 Institute of Bioinformatics and Biosignal Transduction, College of Bioscience and Biotechnology, National Cheng Kung University, Tainan, Taiwan; 3 Institute of Biological Chemistry, Academia Sinica, Taipei, Taiwan; 4 The Ph.D. Program for Translational Medicine, College of Medical Science and Technology, Taipei Medical University and Academia Sinica, Taipei, Taiwan; 5 Graduate Institute of Cancer Biology and Drug Discovery, College of Medical Science and Technology, Taipei Medical University, Taipei, Taiwan; Hosei University, JAPAN

## Abstract

DNA mimicry is a direct and effective strategy by which the mimic competes with DNA for the DNA binding sites on other proteins. Until now, only about a dozen proteins have been shown to function via this strategy, including the DNA mimic protein DMP19 from *Neisseria meningitides*. We have shown previously that DMP19 dimer prevents the operator DNA from binding to the transcription factor NHTF. Here, we provide new evidence that DMP19 monomer can also interact with the *Neisseria* nucleoid-associated protein HU. Using BS3 crosslinking, gel filtration and isothermal titration calorimetry assays, we found that DMP19 uses its monomeric form to interact with the *Neisseria* HU dimer. Crosslinking conjugated mass spectrometry was used to investigate the binding mode of DMP19 monomer and HU dimer. Finally, an electrophoretic mobility shift assay (EMSA) confirmed that the DNA binding affinity of HU is affected by DMP19. These results showed that DMP19 is bifunctional in the gene regulation of *Neisseria* through its variable oligomeric forms.

## Introduction

Protein-DNA interactions are very important to the control of cellular mechanisms such as gene regulation, DNA repair and recombination [1]. DNA mimic proteins are one of the emerging control factors that affect these interactions by occupying the DNA binding sites on the proteins [1–3]. Control factors of this category usually present with DNA phosphate backbone-like negative surface charge distributions and DNA double helical groove-like shapes to deceive the DNA binding proteins [1–3]. However, fewer than 20 DNA mimic proteins have been reported so far, and much more basic research is still needed to fully understand the novel mechanisms of these control factors [3].

In our previous attempts at discovering novel DNA mimic proteins [3–9], *Neisseria meningitides* DMP19 (DNA mimic protein 19 kDa) was one of the DNA mimic proteins that we identified [5]. The crystal structure of the DMP19 dimer has a B-form DNA-like negative charge distribution on one side of its surface. This specialized pattern of negative charges is also complementary to the positive charge distribution of the DNA-binding region of the *Neisseria* transcription factor NHTF [5]. Functionally, the DMP19 dimer prevents NHTF from binding to DNA by directly occupying its DNA-binding site [5]. In the present study, we now show that DMP19 in its monomeric form can also interact with the *Neisseria* nucleoid-associated protein HU dimer. We first observed this interaction in a BS3 crosslinking assay, and the complex formed by the DMP19 monomer and HU dimer was then identified by gel filtration. The binding affinity between these two proteins was determined using isothermal titration calorimetry (ITC), and the calculated binding stoichiometry suggested that the ratio of DMP19 monomer to HU dimer was 1:1 in this complex. Cross-linking coupled mass spectrometry was then used to predict the interface of the binding. Finally, an electrophoretic mobility shift assay (EMSA) confirmed that the DNA binding affinity of HU was attenuated by DMP19. All these results suggest that DMP19 monomer is a competitive inhibitor of the *Neisseria* HU protein. We therefore conclude that DMP19 is a bi-functional DNA mimic protein that is involved in controlling nucleoid formation as well as gene regulation.

## Materials and methods

### Preparation and purification of recombinant DMP19 and *Neisseria* HU protein

The purification of DMP19 and *Neisseria* HU was carried out as described previously [5–6]. Briefly, both proteins were expressed at 16°C for 16 hours after the addition of 1 mM isopropyl-β-D-thiogalactopyranoside (IPTG). Immobilized metal-ion chromatography (Ni-NTA) and gel filtration (Superdex 200 pg) were used to purify the soluble proteins. The proteinase FactorXa was used to remove the N-terminal His_10_-tag of DMP19.

### Bissulfosuccinimidyl suberate (BS3) cross-linking assay of DMP19 and *Neisseria* HU

For the cross-linking assay, C-terminal His_6_-tagged *Neisseria* HU and DMP19 were both dialyzed to 1X PBS buffer. Subsequently, 30 μM purified C-terminal His_6_-tagged *Neisseria* HU protein was mixed with 30 μM DMP19 in 100 μl binding buffer (1x PBS), and the reaction mixture was incubated for 15 minutes at 25°C. Following the addition of BS3 (Sigma-Aldrich) to a final concentration of 0.5 mM, incubation was then continued for another 30 min. Each reaction was stopped by 50 mM Tris-HCl. Finally, 6 μl of each reaction mixture were analyzed by SDS-PAGE.

### His-pulldown assay of DMP19 and *Neisseria* HU

A His-pulldown assay was used to confirm the interaction between DMP19 and *Neisseria* HU. Briefly, 30 μM C-terminal His_6_-tagged *Neisseria* HU and 30 μM DMP19 were mixed and incubated in 100 μl binding buffer (1x PBS; 20mM imidazole) for 15 minutes at 25°C. After adding 20 μl magnetic Ni-NTA beads (Genscript), the mixture was incubated for another 15 minutes. Subsequently, the beads were collected by a magnetic stand and washed with 1 ml binding buffer three times. Finally, the proteins that bound to the beads were eluted by elution buffer (20 mM Tris base pH8.0, 100 mM NaCl and 500 mM imidazole) and analyzed by SDS-PAGE.

### Determination of the native molecular weights by gel filtration

A Superose 12 gel filtration column (GE Healthcare) was used to evaluate the approximate molecular weight of the DMP19 and HU complex. The buffer used in this assay was 20 mM Tris pH 7.4 and 100 mM NaCl. For the sample loading, either 100 μl of sample buffer containing 750 μM C-terminal His_6_-tagged HU or 1500 μM tag-free DMP19 were used, or both 750 μM C-terminal His_6_-tagged *Neisseria* HU and 1500 μM tag-free DMP19 together. Four standard proteins Conalbumin (C; 75 kDa) ovalbumin (OA; 43 kDa), carbonic anhydrase (CA; 29 kDa), and RNase A (RA; 13.7 kDa) were also analyzed by the same column to obtain a plot of Kav against log MW. For each protein, the logarithm of molecular weight (log MW) was plotted against Kav, which was calculated as follows: Kav = (Ve -Vo)/(Vt -Vo), where Ve is the elution volume, Vo is the column void volume using blue dextran 2000, and Vt is the total column bed volume (24 ml for the Superose 12 10/30 gel filtration column).

### Determination of the binding affinity between DMP19 and *Neisseria* HU using isothermal titration calorimetry (ITC)

ITC200 (GE Healthcare) was used to measure the binding affinity of HU and DMP19. Before the titration, both HU and DMP19 were dialyzed against an ITC buffer of 20 mM Tris pH 7.4 and 50 mM NaCl. After the buffer change, the C-terminal His_6_-tagged *Neisseria* HU dimer (300 μM) and DMP19 monomer (30 μM) were loaded into the syringe and sample cell, respectively. Two μl of the HU was injected every 3 min until the DMP19 was saturated (a total of 20 injections) at 25°C. Except for the first injection point, data from the entire titration curve were used to calculate the thermodynamic parameters. The program ORIGIN 7 (GE Healthcare) was used to calculate enthalpy (*ΔH*), binding entropy (*ΔS*), the equilibrium constant (1/*K*_*d*_) and the stoichiometric ratio (N). Gibbs free energy (*Δ*G) was calculated using the equation: *Δ*G = *Δ*H − T*Δ*S.

### Cross-linking coupled mass spectrometry

The experimental procedure was modified from a crosslinking based LC/MS study [10]. Briefly, the bands containing cosslinked DMP19 and HU proteins were excised from the SDS PAGE, followed by in-gel digestion with trypsin and chymotrypsin. Subsequently, the digested peptide mixtures were subjected to a NanoLC-nanoESI-MS/MS analysis using the standard protocol of the Academia Sinica Common Mass Spectrometry Facilities of Institute of Biological Chemistry [11–12]. Finally, all data was analyzed by using the Massmatrix software [13].

### Construction of a binding model for DMP19 and *Neisseria* HU

The crystal structure of the DMP19 monomer (PDB ID: 3WUR) and a protein model of the *Neisseria* HU dimer were used to make the DMP19-HU complex. The three-dimensional structure of *Neisseria* HU dimer was predicted using SWISS-MODEL Web server (http://swissmodel.expasy.org/) with a HU dimer of *Anabaena* HU-DNA cocrystal structure (PDB ID: 1P78) as the modelling template. The DNA binding region of *Neisseria* HU was also proposed by analogy to the *Anabaena* HU-DNA cocrystal structure (S1 Fig). Subsequently, the crystal structure of DMP19 monomer and the predicted model of *Neisseria* HU dimer were uploaded to the ZDOCK server (http://zdock.uma2ssmed.edu) [14] to generate potential DMP19-*Neisseria* HU complexes. A complex that satisfied the cross-linking distance from the output complexes was selected for further analysis. The graphic software Chimera was used to produce the figures in this study [15].

### DNA binding assays (EMSA assays)

To observe the HU/DNA binding, 8 μM C-terminal His_6_-tagged *Neisseria* HU protein dimer (16 μM monomer) was mixed with 0.5 μM double-stranded DNA substrate (48 bp; 5’- CTC GCG AGC TCG CGA GCT CGC GAG CTC GCG AGC TCG CGA GCT CGT CGC GAG—3’) in 20 μl reaction buffer (20 mM Tris pH 7.4 and 100 mM NaCl) and incubated at 25°C for 15 min. For the competitive binding assays, 8 μM His_6_-tagged HU dimer was mixed with 8 or 16 μM DMP19 in 14 μl reaction buffer and pre-incubated at 25°C for 30 min. Subsequently, 1 μl of binding buffer containing the DNA substrate was added, and the incubation continued for 15 min. The reaction products were analyzed by a 4% agarose gel and stained with SYBR Green I (Sigma-Aldrich).

## Results

### Evidence of an interaction between the DNA mimic protein DMP19 and the DNA binding protein *Neisseria* HU

**Fig 1:**
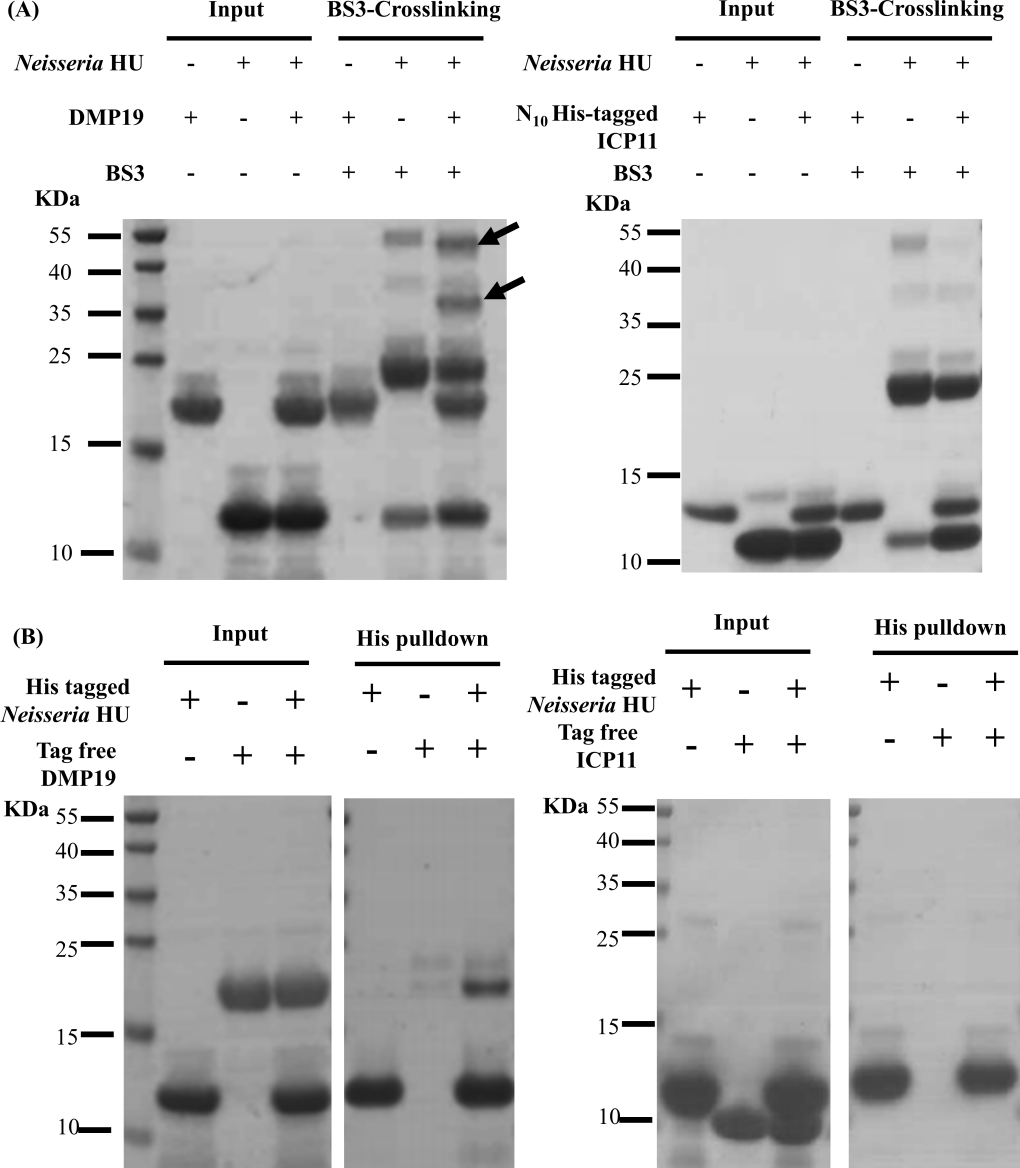
BS3 cross-linking assay and His-pulldown assays confirmed an interaction between DMP19 and *Neisseria* HU. (A) BS3 cross-linking assay. The interaction between DMP19 and *Neisseria* HU was first seen in a BS3 cross-linking assay, which was carried out to screen for interactions between DNA mimic proteins and DNA binding proteins. The shifted bands that indicated cross-linked DMP19 and *Neisseria* HU proteins are labeled with arrows. In a control reaction, another DNA mimic protein, ICP11, was used to replace DMP19 in the BS3 cross-linking assay. No significant cross-linked ICP11 and HU was found. (B) His pull-down assay. The tag -free DMP19 could be pulled-down by C-terminal His_6_-tagged *Neisseria* HU. By contrast, the tag-free ICP11 was not found in the pull-down result.

**Fig 2:**
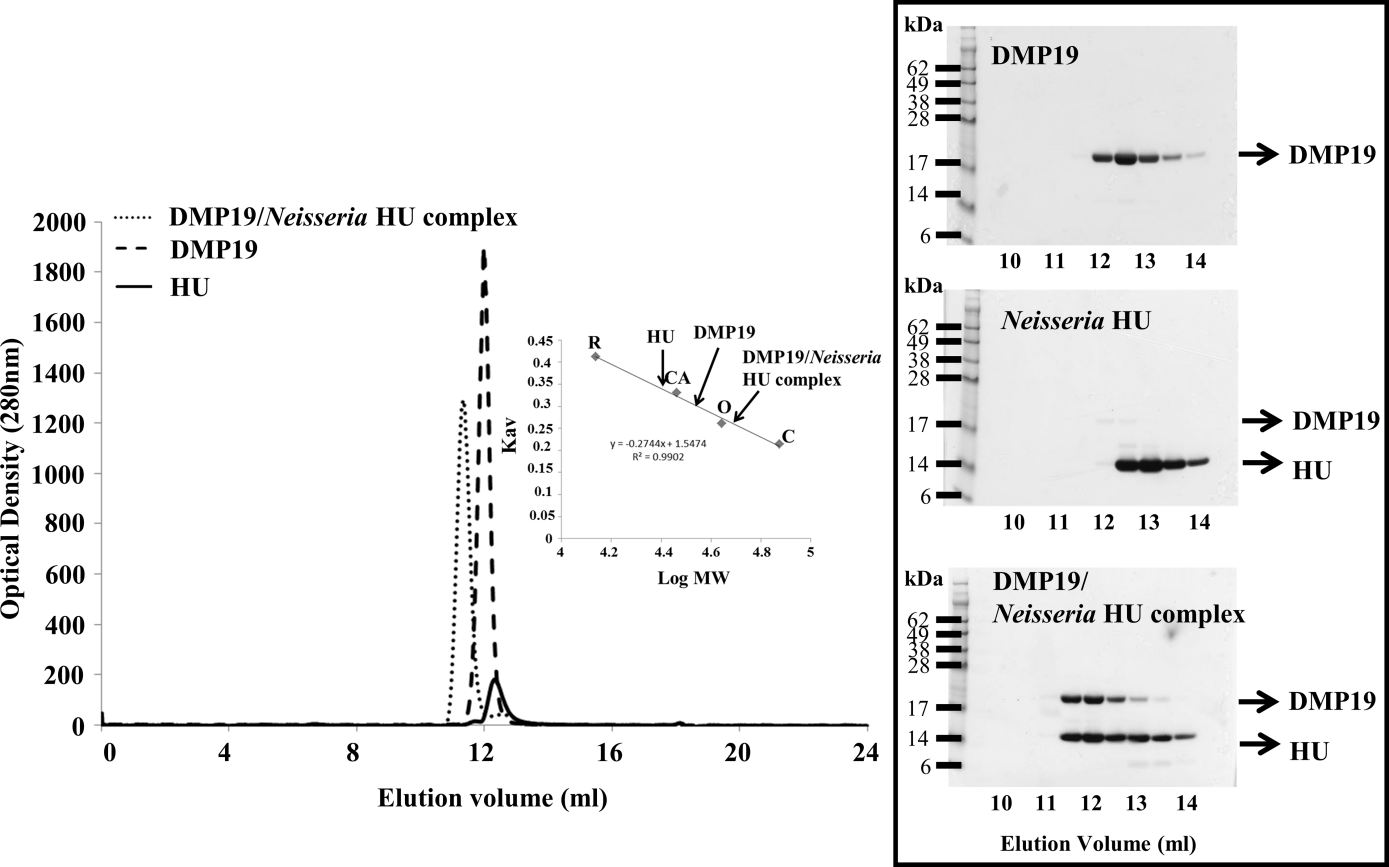
The native molecular weights of DMP19, *Neisseria* HU and DMP19/ *Neisseria* HU complex were calculated by gel filtration. Four standard proteins Conalbumin (C; 75 kDa) ovalbumin (OA; 43 kDa), carbonic anhydrase (CA; 29 kDa), and RNase A (RA; 13.7 kDa) were used to produce a plot of Kav against log MW. The Kav of each target protein was then used to estimate its molecular weight (Table 1).

When we used BS3 crosslinking assays to search for new interactions between DNA mimic proteins and DNA binding proteins, we found that DMP19 could interact with *Neisseria* HU (Fig 1A). Shifted bands that indicated the cross-linking of proteins were observed only in the *Neisseria* HU/DMP19 reaction (Fig 1; arrows). An interaction between *Neisseria* HU and DMP19 was further confirmed by a His-pulldown assay (Fig 1B). We next used gel filtration to estimate the native molecular weights of DMP19, *Neisseria* HU, and the complex (Fig 2; Table 1). Consistent with our previous reports [5–6], the observed molecular weights of both the DMP19 and *Neisseria* HU proteins were close to the theoretical molecular weights of their dimeric forms in the gel filtration analysis (Table 1). However, the observed molecular weight of the DMP19/*Neisseria* HU complex was about 45.7 kDa, which is close to both of the possible monomer/dimer interactions but does not match either the monomer/monomer or dimer/dimer interaction (Fig 2; Table 1). This suggests the monomeric form of one of the two proteins interacts with the dimeric form of the other protein.

**Table 1:** A summary of the gel filtration results.

	Theoretical MW	MW as measured from the Superose 12 column
**DMP19**		
DMP19 (tag free) monomer	18.5 kDa	33.1 kDa
DMP19 (tag free) dimer*	37.0 kDa	
***Neisseria* HU (C-His**_**6**_**)**		
*Neisseria* HU (C-His_6_) monomer	10.4 kDa	27.5 kDa
*Neisseria* HU (C-His_6_) dimer*	20.8 kDa	
**DMP19/*Neisseria* HU (C-His**_**6**_**) complex**		
DMP19 monomer to HU monomer	28.5 kDa	45.7 kDa
DMP19 dimer to HU monomer*	47.8 kDa	
DMP19 monomer to HU dimer*	39.3 kDa	
DMP19 dimer to HU dimer	57.8 kDa	

*Asterisks indicate theoretical values that are consistent with the observed molecular weight (MW).

### DMP19 monomer binds to the *Neisseria* HU dimer with 1:1 stoichiometry

**Fig 3:**
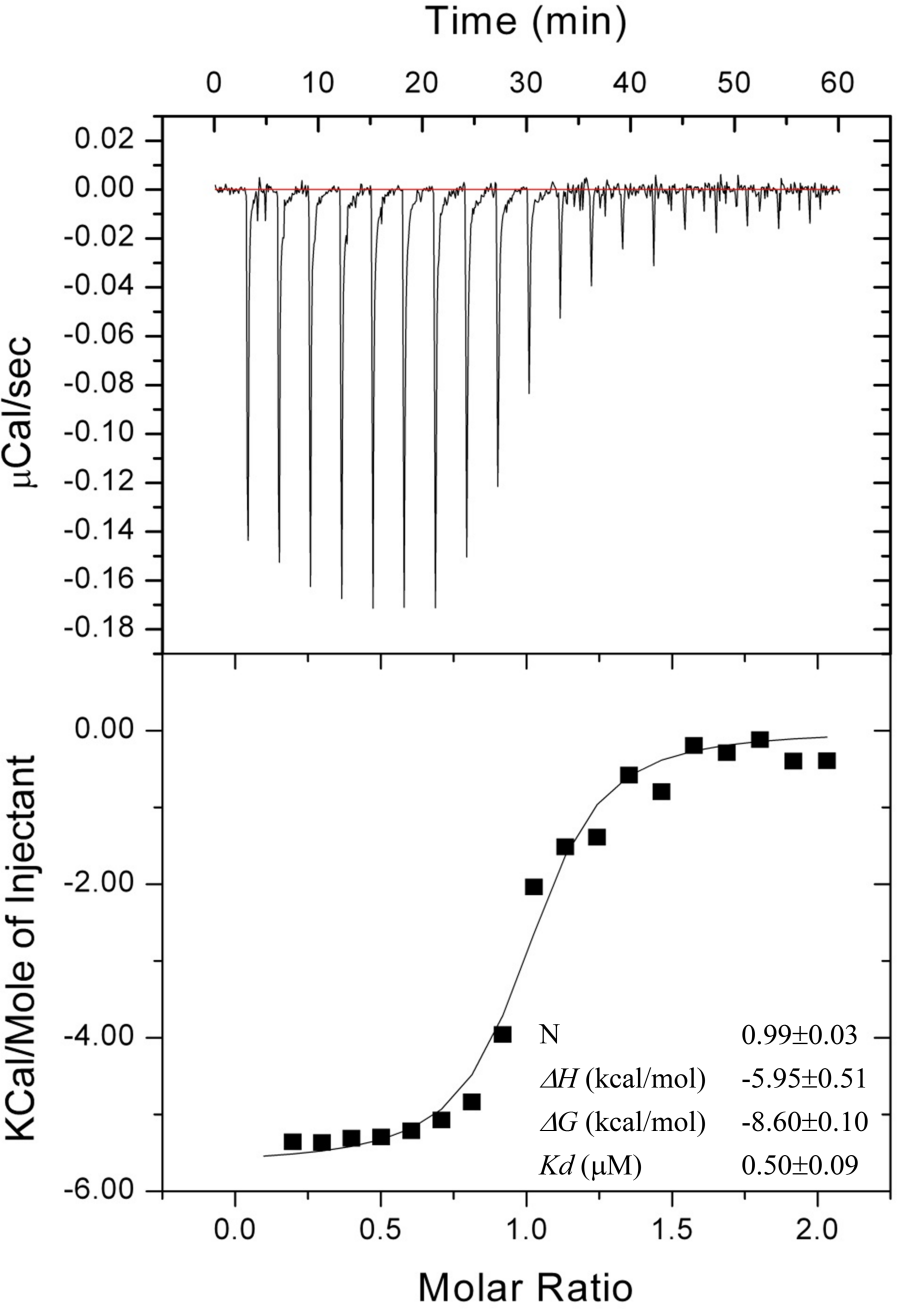
An isothermal titration calorimetry (ITC) assay for the binding affinity between DMP19 and *Neisseria* HU protein. The equilibrium dissociation constant (Kd) of DMP19 monomer and *Neisseria* HU dimer was 0.50 ± 0.09 μM from three replicate experiments. Detailed thermodynamic parameters are shown in Table 2.

Isothermal titration calorimetry (ITC) was used to determine both binding affinity as well as stoichiometry of *Neisseria* HU and DMP19. When 300 μM HU dimer was titrated into 30 μM DMP19 monomer, a 1:1 binding ITC curve could be seen (stoichiometry; N = 1) (Fig 3; Table 2). This result was consistent with a *Neisseria* HU dimer/DMP19 monomer binding. The binding constant (Kd) is 0.50 ± 0.09 μM. Compared to the binding of *Neisseria* HU dimer to 8-mer DNA (Kd = 0.72 ± 0.06 μM, which was also determined by ITC; reference 6), DMP19 monomer has a relatively higher affinity to the HU dimer.

**Table 2:** Thermodynamic parameters for interactions between *Neisseria* HU dimer and DMP19 monomer.

HU dimer to DMP19 monomer	delta H (kcal/mol)	delta G (kcal/mol)	N	Kd (uM)
1^st^ titration	-6.72	-8.4494	0.95	0.64
2^nd^ titration	-5.48	-8.753	1.04	0.38
3^rd^ titration	-5.65	-8.60514	0.98	0.49
AVG with STDEV	-5.95±0.52	-8.60±0.10	0.99±0.03	-0.50±0.09

### Crosslinked pairs of lysine residues lead to a binding model in which DMP19 occupies the DNA binding site on *Neisseria* HU dimer

**Fig 4:**
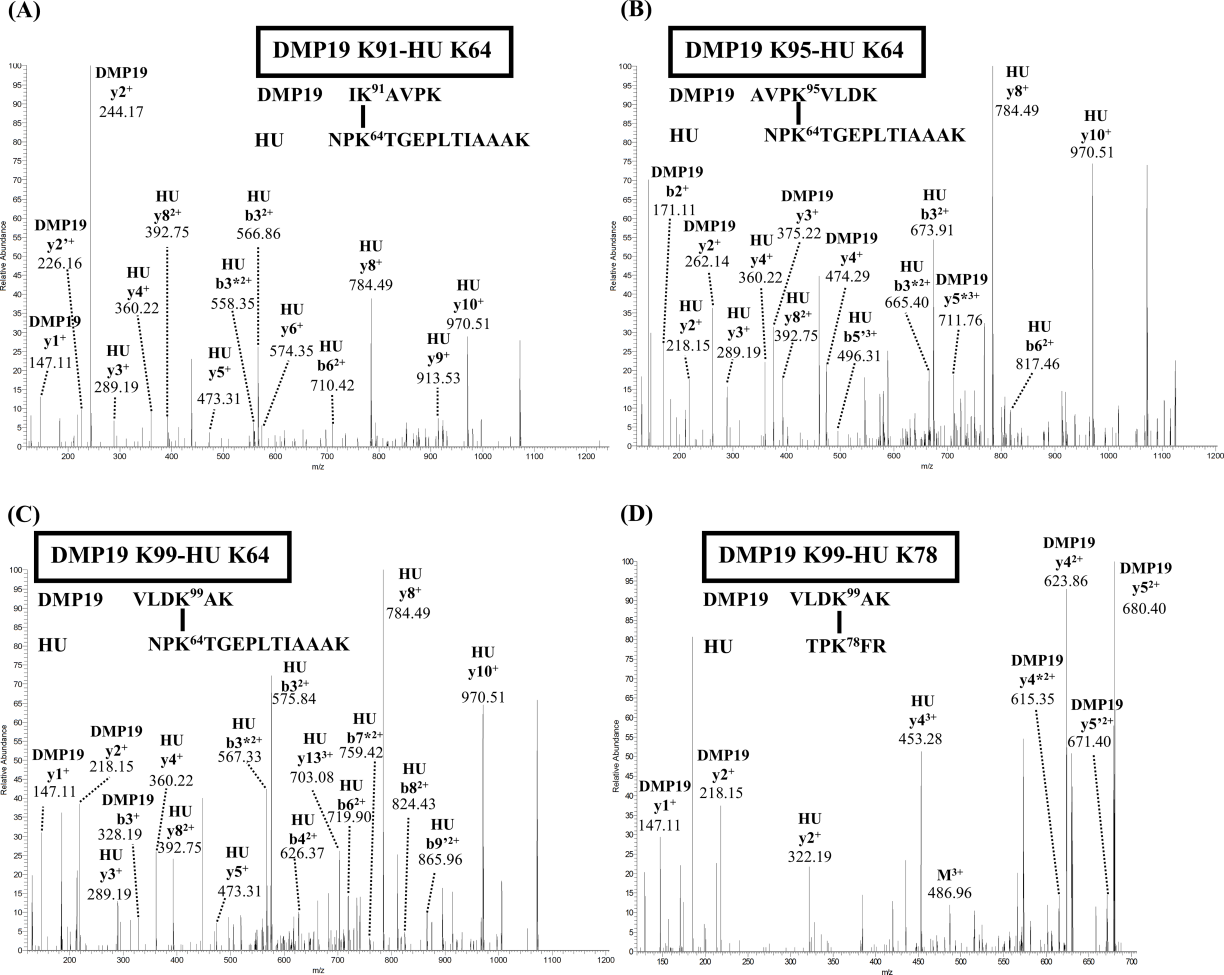
The MS/MS spectra of crosslinked lysine residues between DMP19 and *Neisseria* HU. (A) DMP19 K91 to HU K64, (B) DMP19 K95 to HU K64, (C) DMP19 K99 to HU K64, and (D) DMP19 K99 to HU K78.

**Fig 5:**
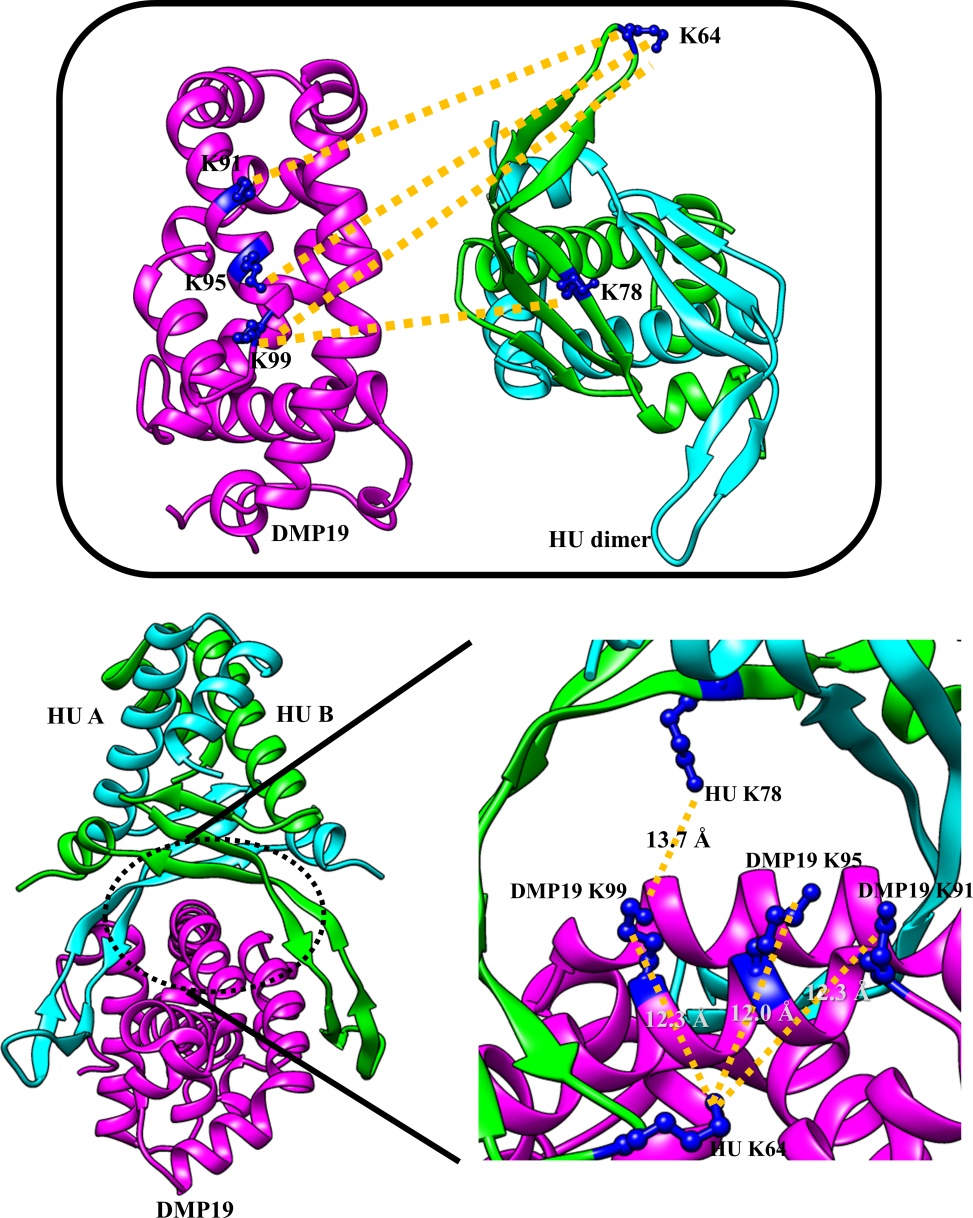
A proposed binding model of DMP19 and *Neisseria* HU. The model was constructed based on the crosslinked lysine residues of DMP19 monomer (crown ether conformation; see S2 Fig) and *Neisseria* HU dimer (top panel). The distances (Å) between the matched pairs of crosslinked lysine residues are shown.

Cross-linking coupled mass spectrometry was used to study the binding mode between DMP19 monomer and HU dimer. The crosslinked DMP19/*Neisseria* HU proteins in the shifted bands (Fig 1; arrows) were extracted and analyzed by nanoLC-nanoESI-MS/MS. The results showed four crosslinked pairs between DMP19 lysine residues of DMP19 (Lys 91, Lys 95 and Lys 99) and HU (Lys 64 and Lys 78; Fig 4; Table 3). We next constructed a binding model of the DMP19 monomer and HU dimer based on these observations. Given that the end-to-end distance of the crosslinker BS3 is 11.4 Å, three lysine residues on DMP19 (Lys 91, Lys 95 and Lys 99) can be connected with the Lys 64 of one of the monomers in the the HU dimer, while DMP19 Lys 99 can also connect with the Lys 78 of the same HU monomer. By adjusting the orientations and positions of the DMP19 monomer and *Neisseria* HU dimer so that the distances between the connected Lys residues are all close to 11.4 Å, we constructed the three-dimensional model shown in Fig 5. In this model, the DMP19 monomer directly occupies the DNA-binding region of *Neisseria* HU dimer and thus prevents its binding to DNA.

**Table 3:** Identified crosslinked peptides of the DMP19 and HU proteins.

Crosslinked Lysine residues	PP / PP2 / PPtag score	MW (obs) (Da)	MW (Da)	Assigned peptide sequence	
				DMP19 peptide (Chain A)	HU peptide (Chain B)
**DMP19 K91-HU K64**	15.3/14.4/1.9	1491.9128	1491.9006	IK(91)AVPK	QGRNPK(64)
	18.9/15.2/1.6	1491.9144	1491.9006	IK(91)AVPK	QGRNPK(64)
	44.5/23.1/7.6	2203.3059	2203.3060	IK(91)AVPK	NPK(64)TGEPLTIAAAK
	41.8/25.7/9.2*	2203.3094	2203.3060	IK(91)AVPK	NPK(64)TGEPLTIAAAK
	17.9/18.5/7.3	2814.6548	2814.6591	IK(91)AVPK(95)VL DK(99)	NPK(64)TGEPLTIAAAK
**DMP19 K95-HU K64**	24.6/25.2/10.4	2772.6092	2772.6121	AVPK(95)VLDK(100) AK(101)	NPK(64)TGEPLTIAAAK
	23.7/23.8/7.7	2772.6123	2772.6121	AVPK(95)VLDK(100) AK(101)	NPK(64)TGEPLTIAAAK
	30.0/26.1/6.8	2417.3996	2417.4014	AVPK(95)VLDK	NPK(64)TGEPLTIAAAK
	37.4/26.3/6.9*	2417.4014	2417.4014	AVPK(95)VLDK	NPK(64)TGEPLTIAAAK
**DMP19 K99-HU K64**	39.2/28.5/4.0	2221.2812	2221.2802	VLDK(99)AK	NPK(64)TGEPLTIAAAK
	49.0/31.6/7.4*	2221.2821	2221.2802	VLDK(99)AK	NPK(64)TGEPLTIAAAK
**DMP19 K99-HU K78**	36.0/24.9/3.2	1458.8673	1458.8679	VLDK(99)AK	TPK(78)FR
	39.5/25.4/3.2*	1458.8694	1458.8679	VLDK(99)AK	TPK(78)FR

* The pattern of this MS/MS analysis was shown in Fig 4.

### DMP19 disrupts DNA binding to *Neisseria* HU dimer

**Fig 6:**
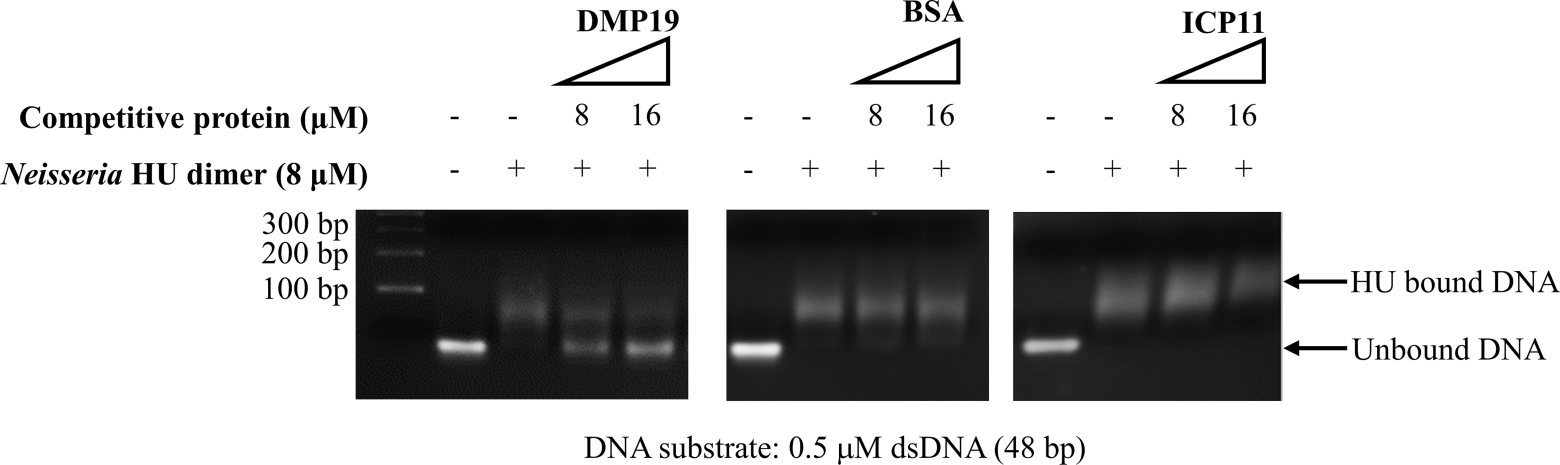
DMP19 prevents the DNA binding of *Neisseria* HU protein. When the molar ratio of DMP19 monomer to HU dimer was 1:1, the DNA shift induced by *Neisseria* HU was reduced by DMP19. No effect was seen for BSA and ICP11.

The above results suggest that DMP19 monomer is likely to be a competitive inhibitor of *Neisseria* HU, and we next therefore used an electrophoresis mobility shift assay (EMSA) to investigate if DMP19 monomer could indeed prevent DNA from binding to *Neisseria* HU dimer. As shown in Fig 6, the DNA band shifting, which indicates the *Neisseria* HU-DNA binding, was significantly decreased by adding DMP19 monomer into the reaction. By contrast, neither of the negatively charged control proteins, BSA (Bovine serum albumin) and ICP11 (another DNA mimic that binds histone), caused any change in the EMSA band position of the HU-bound DNA. This result supports the above model in which the DMP19 monomer directly occupies the DNA-binding region of HU dimer, and it confirmed the strong interaction between the two proteins.

## Discussion

*Neisseria* DMP19 is one of the DNA mimic proteins that we recently identified [5]. In the first report of *Neisseria* DMP19, this DNA mimic was proposed as a transcriptional regulator [5]. The crystal structure showed that DMP19 dimer has a surface charge distribution that is complementary to the DNA binding surface of *Neisseria* NHTF transcription factor, and thus affects the binding of NHTF to its respective operator DNA [5]. Functionally, NHTF belongs to the XRE (Xenobiotic Response Element) transcription factor family and acts as an auto-feedback repressor of the down-stream genes including *NHTF* itself and the Protein-PII uridylyltransferase gene *glnD* [5]. The binding of DMP19 dimer to NTHF interferes with this negative gene control, and restores the expression of the affected genes [5].

In a subsequently determined DMP19 structure, a new conformation was revealed when crown ether was present in the crystal droplet [16]. In this new DMP19 structure, helices α1 and α2 were rotated by ~180 degrees relative to the original structure (S2 Fig). Although two DMP19 monomers were found in an asymmetric unit of the crystal, two intercalating crown ether molecules prevented formation of the core DMP19 dimer interface (S2 Fig; right). Furthermore, the rotated helices α1 and α2 prevented these two monomers from coming close enough together to assemble into a dimer. These observations suggest that DMP19 is monomeric in this new crystal form. Interestingly, when we tried to dock DMP19 monomer and *Neisseria* HU dimer, helices α1 and α2 of the DMP19 monomer from the first crystal of dimeric DMP19 produced a steric clash with the HU protein, and only the DMP19 monomer from the new crystal could be successfully docked onto the HU dimer (Fig 5). All of these observations suggest that DMP19 has two conformations as well as two oligomeric states and that these are used to perform different functions. Thus in addition to binding to NHTF as a dimer [5], here we provide evidence that DMP19 has another functional role as a monomer, specifically, that it could act as a competitive regulator of *Neisseria* HU.

The highly-conserved bacterial nucleoid-associated HU proteins are involved in many important functions such as nucleoid compaction, gene regulation and DNA replication initiation [17]. HU proteins usually function as a dimer. They induce DNA bending that results in a consistent negative supercoiling via non-sequence-specific DNA binding [18–19]. In addition to DNA, HU is also capable of binding to RNA as well as DNA-RNA hybrids [20]. By binding to rpoS mRNA, *Escherichia* HU is able to regulate rpoS translation [21]. Furthermore, it has recently been confirmed that HU can interact with noncoding RNAs in *E*. *coli*. [22–23], and that its homodimeric and heterodimeric forms (i.e. HUα/HUα and HUα/HUβ) control nucleoid compaction in *E*.*coli* [24]. Although the nucleic acid binding affinity of HU protein has been investigated in detail, only a limited number of reports have studied the protein network between HU and its control proteins. Nevertheless, protein-protein interactions with HU are clearly important. For example, an interaction between DnaA and HU is critical for initiation at the replication origin of the *E*. *coli* chromosome [25].

**Fig 7:**
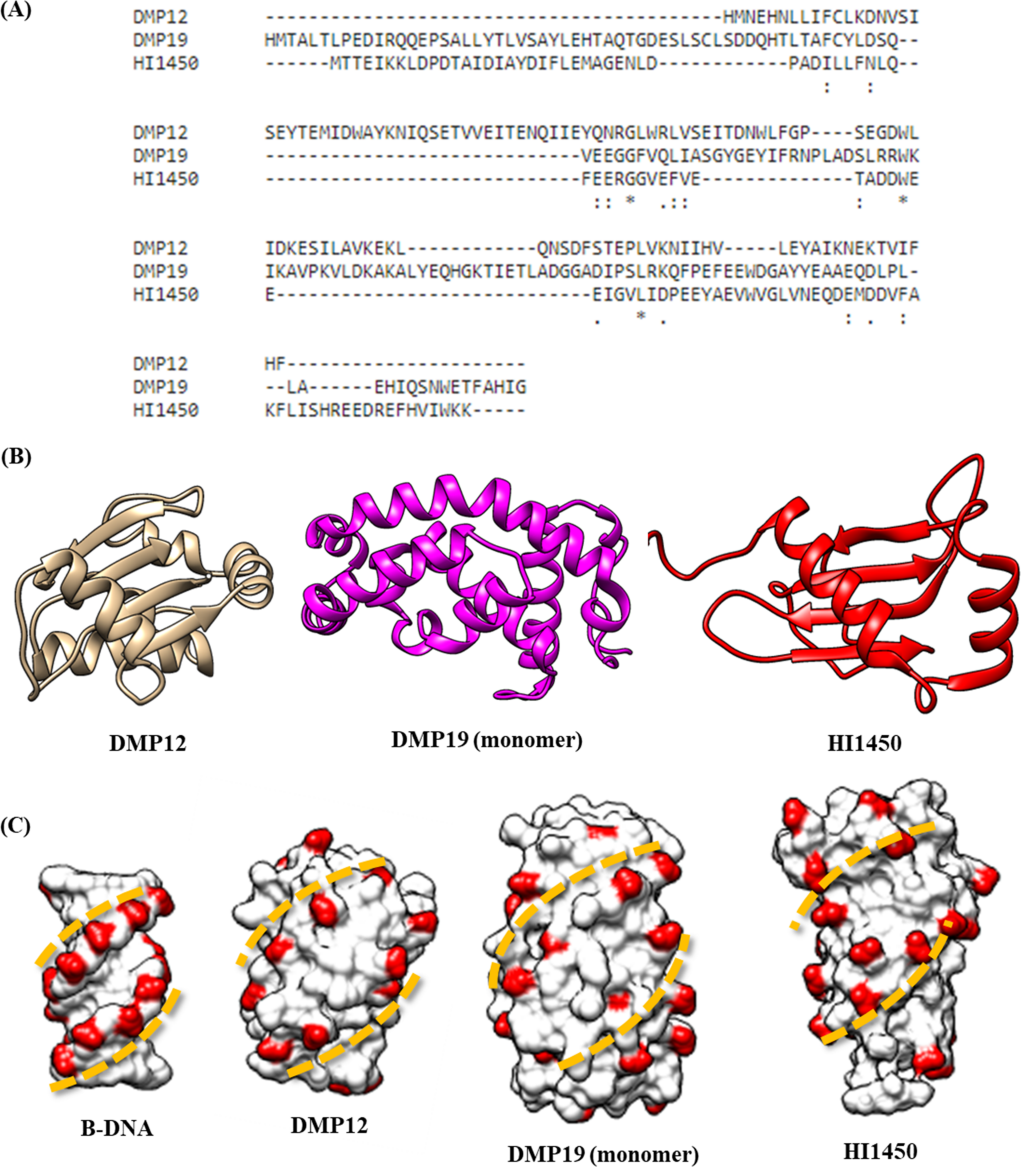
Comparison of three HU-binding DNA mimic proteins. (A) Amino acid alignment of DMP19, DMP12 and HI1450. This analysis was performed by Cluster omega (https://www.ebi.ac.uk/Tools/msa/clustalo/). (B) The structures of DMP19, DMP12 and HI1450. (C) The surface negative charge distributions of DMP19, DMP12 and HI1450. The HU-bound surface of the DMP19 monomer as determined by crosslinking-based mass spectrometry contains two rows of negative charge distributions. This specialized charge arrangement is similar to that of B-form DNA, and it can also be found in the other two DNA mimic proteins. The PDB IDs used to produce this figure are: 1NNV, 3W1O, 3WUR and 1ZEW for HI1450, DMP12, DMP19 and B-form DNA, respectively.

In addition to DMP19, two other DNA mimic proteins, *Neisseria* DMP12 and *Haemophilus influenzae* HI1450, have also been shown to bind to HU proteins [6, 26–27]. All three of these DNA mimic proteins were found to bind to HU protein in their monomeric form, but since the amino acids and structures of DMP12, DMP19 and HI1450 are all different to each other (Fig 7A and 7B), it is surprising that these three DNA mimic proteins should be able to bind to the same target. However, we note that they all share a similar B form DNA-like negative charge distribution on their surface (Fig 7C), and these specialized negative charge distributions may play an important role in the recognition of HU proteins. The HU binding activities of these three DNA mimic proteins have all been confirmed by ITC assays [6, 27]. Compared to DMP12 and HI1450, DMP19 has the highest binding affinity to HU proteins (Kd = 2.8 and 0.5 μM for DMP12 and DMP19 monomer to *Neisseria* HU binding; 3.0 μM for H1450 to *Haemophilus influenzae* HU; all measured by ITC). Among these three DNA mimics, only DMP19 monomer has a higher binding affinity to *Neisseria* HU than 8bp dsDNA (Kd = 0.7 μM; reference 6), suggesting DMP19 monomer can serve as a potent inhibitor of *Neisseria* HU.

The next question to consider is how DMP19 might use its bi-functional activity to carry out transcriptional control. We hypothesize that DMP19 is an effective gene enhancer in *Neisseria* spps. By preventing HU from binding, DMP19 monomer may first release selected gene regions in chromosomal DNA. Subsequently, if DMP19 dimer disrupts the gene repressor activity of NHTF, this would ensure the continued expression of NHTF-controlled genes. Thus, for example, *glnD* would still be expressed in response to changes in the environment. However, this hypothesis still needs to be confirmed. Meanwhile, the results presented here provide strong evidence that DMP19 monomer is a competitive inhibitor of *Neisseria* HU, and that DMP19 therefore possesses a bi-functionality that depends on its different oligomeric states. This ability is unique among the few known DNA mimic proteins.

## Supporting information

S1 Fig Proposed *Neisseria* HU/DNA binding model. The Lys 78 residues of the *Neisseria* HU dimer are located at the center of the DNA binding region. Spatially, the Lys 64 residues are located at the ends of the flexible arms. All of these features are important for HU-DNA binding.
(PDF)

S2 Fig Comparison of two different experimentally-confirmed DMP19 structures. The conformational differences shown here may affect the oligomerization and thus the functionality of DMP19.
(PDF)
