# Supplementary material for: The monomeric form of Neisseria DNA mimic protein DMP19 prevents DNA from binding to the histone-like HU protein
Source: PLoS One. 2017 Dec 8;12(12):e0189461. doi: 10.1371/journal.pone.0189461 (PMC5722371; doi:10.1371/journal.pone.0189461)
Supplement: S1 Fig — The Lys 78 residues of the Neisseria HU dimer are located at the center of the DNA binding region. Spatially, the Lys 64 residues are located at the ends of the flexible arms. All of these features are important for HU-DNA binding. (PDF) [file pone.0189461.s001.pdf]

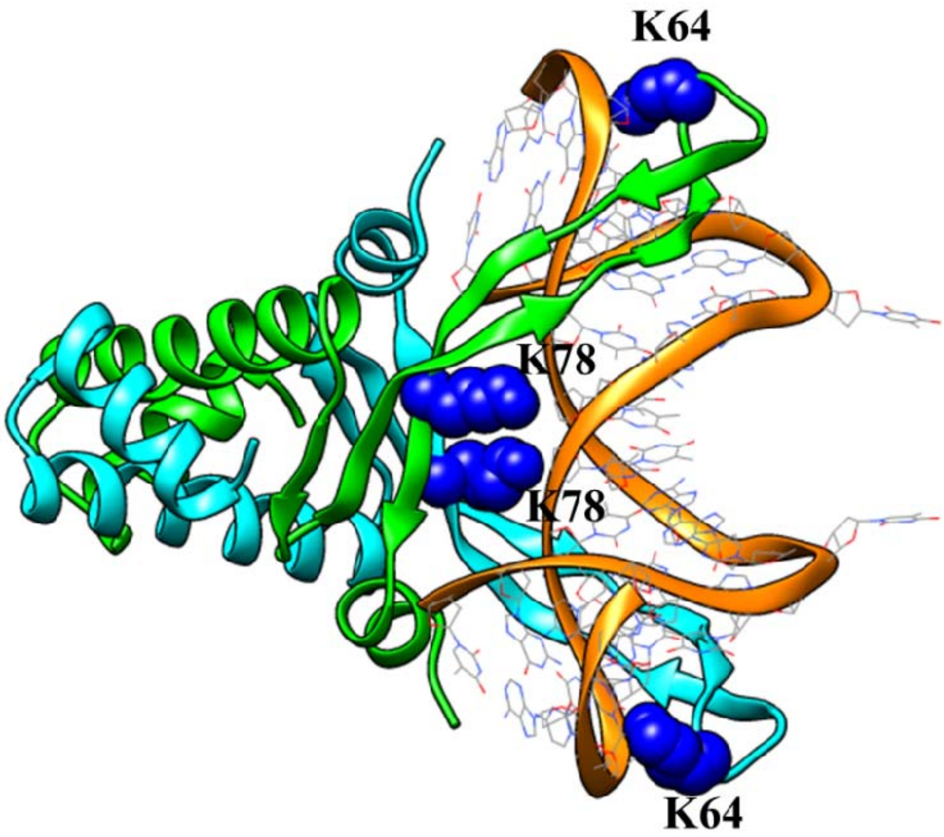

S1 Fig. Proposed *Neisseria* HU/DNA binding model. The Lys 78 residues of the *Neisseria* HU dimer are located at the center of the DNA binding region. Spatially, the Lys 64 residues are located at the ends of the flexible arms. All of these features are important for HU-DNA binding.
