# Supplementary material for: The monomeric form of Neisseria DNA mimic protein DMP19 prevents DNA from binding to the histone-like HU protein
Source: PLoS One. 2017 Dec 8;12(12):e0189461. doi: 10.1371/journal.pone.0189461 (PMC5722371; doi:10.1371/journal.pone.0189461)
Supplement: S2 Fig — The conformational differences shown here may affect the oligomerization and thus the functionality of DMP19. (PDF) [file pone.0189461.s002.pdf]

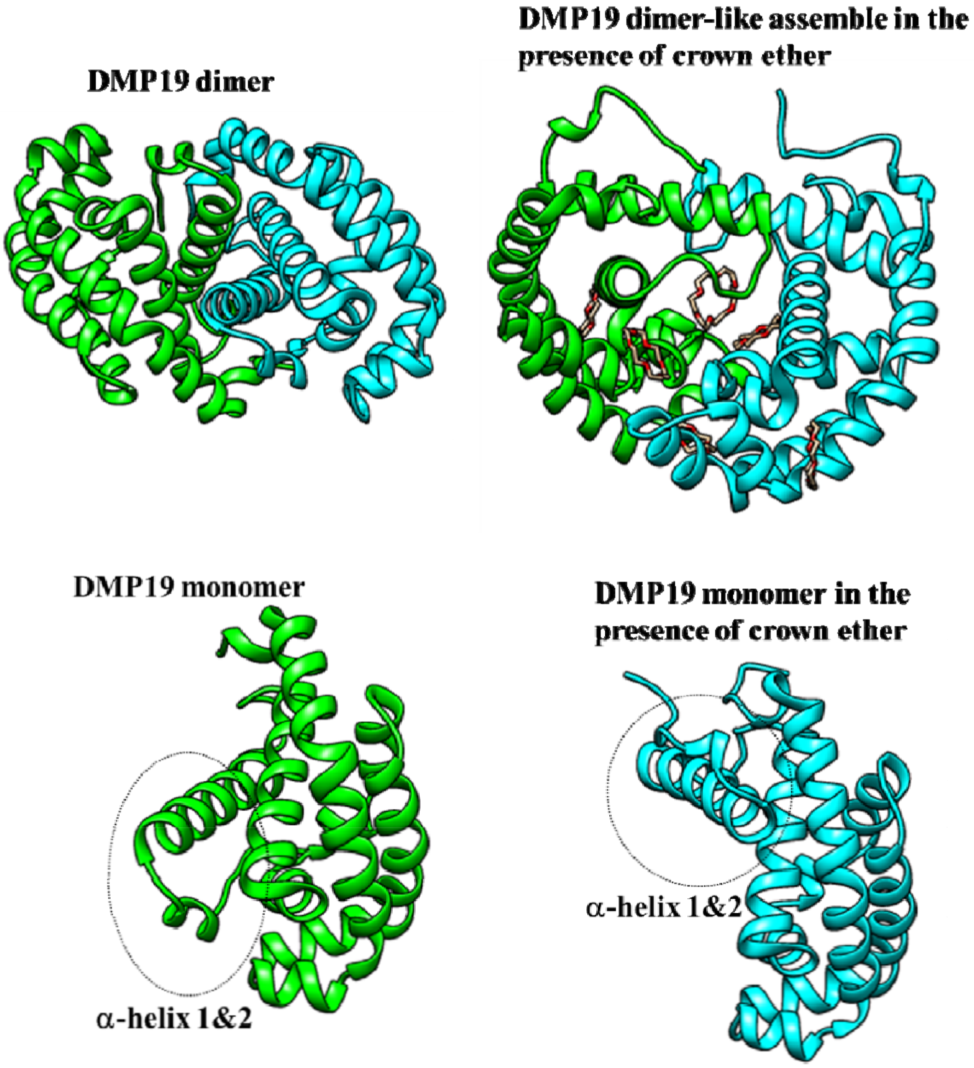

S2 Fig. Comparison of two different experimentally-confirmed DMP19 structures. The conformational differences shown here may affect the oligomerization and thus the functionality of DMP19.
